# Calculation of the total corneal astigmatism using the virtual cross cylinder method on the secondary principal plane of the cornea

**DOI:** 10.1038/s41598-024-55154-x

**Published:** 2024-02-26

**Authors:** Yukitaka Danjo

**Affiliations:** Department of Ophthalmology, Osaka Minato Central Hospital, Japan Community Health care Organization (JCHO), 1-7-1 Isoji, Minato-Ku, Osaka, 552-0003 Japan

**Keywords:** Corneal diseases, Refractive errors

## Abstract

This study aimed to establish a virtual cross cylinder method to calculate the total corneal astigmatism by combining the anterior and posterior corneal astigmatism on the secondary principal plane of the cornea based on Gaussian optics. The meridian with the least refractive power, namely, the flattest meridian of the virtual cross cylinder of a ± 0.5 × C diopter, is set as the reference meridian, and the power (F) at an angle of φ between an arbitrary meridian and the reference meridian is defined as F(φ) =  − 0.5 × C × cos2φ. The magnitude and axis of the total corneal astigmatism were calculated by applying trigonometric functions and the atan2 function based on the combination of the virtual cross cylinders of the anterior corneal astigmatism and the posterior corneal astigmatism. To verify the performance of the virtual cross cylinder method, a verification experiment with two Jackson cross cylinders and a lensmeter was performed, and the measured and calculated values were compared. The limit of the natural domain of the arctangent function is circumvented by using the atan2 function. The magnitude and axis of the total corneal astigmatism are determined through generalized mathematical expressions. The verification experiment results showed good agreement between the measured and calculated values. Compared to the vector analysis method, the virtual cross cylinder method is mathematically sound and straightforward. A novel technique for calculating total corneal astigmatism, the virtual cross cylinder method, was developed and verified.

## Introduction

The refractive power of the cornea, including the tear film, accounts for approximately two-thirds of the total ocular refractive power. Theoretically, the refractive power can be calculated based on Snell’s law with the anterior corneal curve and the refractive indices of air and the cornea. However, accurate measurements of the posterior corneal curve and the refractive index of the total cornea, including the tear film, are difficult to obtain, so a method that does not require them was developed. The K index is a conversion factor that is used to calculate the corneal refractive power based on the anterior corneal curve, and the K index value of 1.3375, the origin of which is unknown, has been traditionally used in most keratometers and is adopted in most contemporary devices, including the IOLMaster700 (Carl Zeiss Meditec). As the use of corneal refractive surgery and premium intraocular lenses increases, the importance of measuring the posterior corneal curve has become increasingly evident^1^. The posterior corneal curve had previously been measured using various methods and instruments, including Purkinje images, Scheimpflug imaging, optical coherence tomography, and other tools^1–3^. The introduction of the swept-source optical coherence technology-based IOLMaster700, which can be used to measure the posterior corneal curve, was expected to decrease postoperative refractive errors in eyes undergoing cataract surgery by using the total keratometry (TK)^4,5^. However, several studies have reported that the refractive performance of TK-based IOL power calculation formulas for normal eyes is not superior to that of K-based formulas^6–8^; in fact, their performance is inferior to that of K-based methods in eyes with relatively normal ranges of axial length and anterior keratometry^8^.

In a previous stage of the present work^9^, the total corneal power (TCP) was computed using two strategies, one with the corneal index n_1_ as the variable and the other the TK index n_x_ as the variable. The virtual axial length (AL) was defined as the distance from the corneal secondary principal plane to the retina. The refractive prediction accuracy of the Barrett Universal II (BUII) formula (unpublished) was evaluated using the refraction results predicted based on the TCP and the virtual AL. The refractive performance of both strategies with the modified BUII formula was better than that of the conventional K-based BUII formula, and the performance was improved further when a TK index of n_x_ = 1.3858 was used in the TK index strategy. The TK index of n_x_ = 1.3858 was determined via the TK index strategy, in which the TK index n_x_ is used as a variable so that the modified BUII formula had the best refractive performance^9^. Thus, I proposed the TK index for TCP calculations. The TK index is related to the total corneal curve, similar to the relation between the K index and the anterior corneal curve.

It has been demonstrated that posterior corneal astigmatism has a significant influence on total corneal astigmatism^1,10,11^. In my previous study, I calculated the TCP with spherical equivalent powers in the anterior and posterior corneal planes^9^. Both planes have steep and flat meridians, namely, astigmatism, and the axes of these meridians are not necessarily the same. In addition, the corneal thickness should be considered in the calculation of the total corneal astigmatism, similar to the TCP calculation. Therefore, the calculation of the total corneal astigmatism is not simple. Traditionally, the total corneal astigmatism has been calculated using the vector analysis method^12–15^. However, this method cannot address, in a mathematically sound way, the case in which the formula has a zero in the denominator or an obtained value of the astigmatic axis is outside the limits. In addition, the results of studies using the vector analysis method to assess anterior corneal astigmatism combined with posterior corneal astigmatism have not been verified experimentally. To calculate the total corneal astigmatism in a mathematically correct manner, a novel technique called the virtual cross cylinder method has been developed and verified in this work.

## Methods

### Definition of the TK plane and descriptive terms

Assume that the anterior corneal astigmatism and the posterior corneal astigmatism are combined in the secondary principal plane of the cornea, which is calculated based on the TK index strategy^9^ and corresponds to the spherical equivalent plane of the total corneal power. I term this plane the TK plane. The following descriptive terms, including K_f_, K_s_, PK_f_, and PK_s_, are derived from keratometry with the IOLMaster700.

- K_f_ : refractive power of the flat meridian of the anterior corneal surface
- K_s_: refractive power of the steep meridian of the anterior corneal surface
- K_f_’: the refractive power of the flat meridian of the anterior corneal surface on the TK plane calculated with the TK index strategy, assuming that the posterior corneal surface has no astigmatism
- K_s_’: the refractive power of the steep meridian of the anterior corneal surface on the TK plane calculated with the TK index strategy, assuming that the posterior corneal surface has no astigmatism
- PK_f_: refractive power of the flat meridian of the posterior corneal surface
- PK_s_: refractive power of the steep meridian of the posterior corneal surface
- PK_f_’: the refractive power of the flat meridian of the posterior corneal surface on the TK plane calculated with the TK index strategy, assuming that the anterior corneal surface has no astigmatism
- PK_s_’: the refractive power of the steep meridian of the posterior corneal surface on the TK plane calculated with the TK index strategy, assuming that the anterior corneal surface has no astigmatism
- n_x_: the TK index
- 1.3375: value of the K index
- 1: refractive index of air
- 1.376: refractive index of the corneal stroma derived from the Gullstrand exact schematic eye
- CCT: central corneal thickness

Note: Since the PK value is negative on the posterior corneal surface^1^ and PK_f_ and PK_s_ are converted to the PK_s_’ and PK_f_’, respectively, on the TK plane, the axis of the posterior corneal astigmatism is reversed on the TK plane.

According to the TK index strategy^9^,

1 $${{\text{K}}}_{{\text{f}}}^{\mathrm{^{\prime}}}=\frac{{{\text{n}}}_{{\text{x}}}-1}{1.3375-1}\times \left({{\text{K}}}_{{\text{f}}}+\frac{{{\text{PK}}}_{{\text{f}}}+{{\text{PK}}}_{{\text{s}}}}{2}-\frac{{\text{CCT}}}{1.376}\times {{\text{K}}}_{{\text{f}}}\times \frac{{{\text{PK}}}_{{\text{f}}}+{{\text{PK}}}_{{\text{s}}}}{2}\right)$$

2 $${{\text{K}}}_{{\text{s}}}^{\mathrm{^{\prime}}}=\frac{{{\text{n}}}_{{\text{x}}}-1}{1.3375-1}\times \left({{\text{K}}}_{{\text{s}}}+\frac{{{\text{PK}}}_{{\text{f}}}+{{\text{PK}}}_{{\text{s}}}}{2}-\frac{{\text{CCT}}}{1.376}\times {{\text{K}}}_{{\text{s}}}\times \frac{{{\text{PK}}}_{{\text{f}}}+{{\text{PK}}}_{{\text{s}}}}{2}\right)$$

3 $${{\text{PK}}}_{{\text{f}}}^{\mathrm{^{\prime}}}=\frac{{{\text{n}}}_{{\text{x}}}-1}{1.3375-1}\times \left(\frac{{{\text{K}}}_{{\text{f}}}+{{\text{K}}}_{{\text{s}}}}{2}+{{\text{PK}}}_{{\text{s}}}-\frac{{\text{CCT}}}{1.376}\times \frac{{{\text{K}}}_{{\text{f}}}+{{\text{K}}}_{{\text{s}}}}{2}\times {{\text{PK}}}_{{\text{s}}}\right)$$

4 $${{\text{PK}}}_{{\text{s}}}^{\mathrm{^{\prime}}}=\frac{{{\text{n}}}_{{\text{x}}}-1}{1.3375-1}\times \left(\frac{{{\text{K}}}_{{\text{f}}}+{{\text{K}}}_{{\text{s}}}}{2}+{{\text{PK}}}_{{\text{f}}}-\frac{{\text{CCT}}}{1.376}\times \frac{{{\text{K}}}_{{\text{f}}}+{{\text{K}}}_{{\text{s}}}}{2}\times {{\text{PK}}}_{{\text{f}}}\right)$$

ACA: the magnitude of the anterior corneal astigmatism on the TK plane

5 $${\text{ACA}}={{\text{K}}}_{{\text{s}}}^{\mathrm{^{\prime}}}-{{\text{K}}}_{{\text{f}}}^{\mathrm{^{\prime}}}$$

PCA: the magnitude of the posterior corneal astigmatism on the TK plane

6 $${\text{PCA}}={{\text{PK}}}_{{\text{s}}}\mathrm{^{\prime}}-{{\text{PK}}}_{{\text{f}}}\mathrm{^{\prime}}$$

TCA: the magnitude of the total corneal astigmatism on the TK plane

### Definition of the virtual cross cylinder

**Figure 1:**
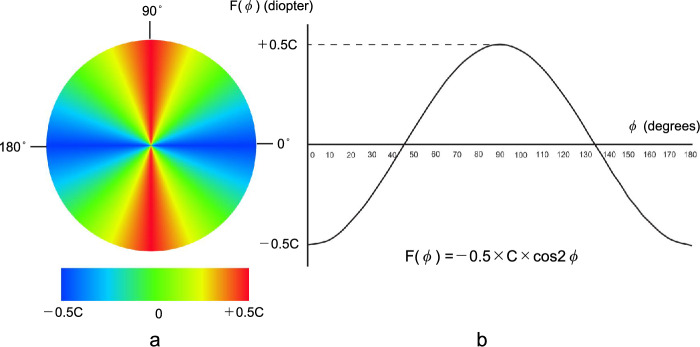
A virtual cross cylinder with a ± 0.5 × C diopter, depicting the power profile with a color coded map (**a**) and a graphical display (**b**) of the power F(φ). *C*: power corresponding to the astigmatic magnitude.

The following formulation is based on the assumption that the anterior corneal astigmatism and posterior corneal astigmatism are both regular astigmatism in the paraxial region. Consider a cross cylinder on the TK plane (Fig. 1a). I term this cross cylinder the “virtual cross cylinder”. The meridian with the least refractive power, namely, the flattest meridian of the virtual cross cylinder, is defined as the reference meridian, the angle of which is defined as zero. The virtual cross cylinder of the ±0.5×C diopter (D) is set to:

7 $$-0.5\times \mathrm{C\ Ax\ }90/+0.5\times \mathrm{C\ Ax\ }180,$$

i.e., in the plus cylinder form,

8 $$\mathrm{Sph }-0.5\times \mathrm{C }=\mathrm{ Cy }+\mathrm{C\ Ax\ }0,$$

as shown in Fig. 1a. The power (F) of the virtual cross cylinder at an angle of φ between an arbitrary meridian and the reference meridian is defined as shown in the following equation:

9 $${\text{F}}\left({\varphi }\right)=-0.5\times {\text{C}}\times {\text{cos}}2{\varphi }$$

$$0^\circ \leq \varphi < 180^\circ (0 \leq \varphi < \pi 
 \mathrm{radian})$$

Although an axis angle of 180 degrees is conventionally used instead of zero degrees, an axis angle of zero degrees is used in this study for mathematical reasons. The results of Equation (9) are plotted in Fig. 1b.

**Figure 2:**
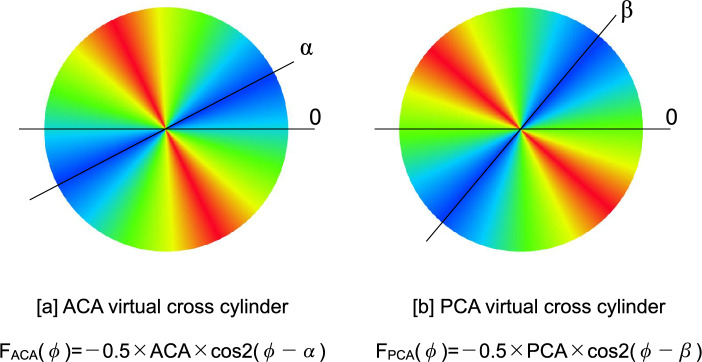
(**a**) A virtual cross cylinder for anterior corneal astigmatism with axis α. The power profile is expressed as F_ACA_(φ) =  − 0.5 × ACA × cos2(φ – α). (**b**) A virtual cross cylinder for posterior corneal astigmatism with axis β. The power profile is expressed as F_PCA_(φ) =  − 0.5 × PCA × cos2(φ – β). *ACA* magnitude of the anterior corneal astigmatism, *PCA* magnitude of the posterior corneal astigmatism.

- The refractive power at an arbitrary angle φ of the ± 0.5 × ACA virtual cross cylinder with an axis angle of α (0 ≤ α < π), corresponding to the angle of K_f_’, can be expressed as (Fig. 2a):
  10 $${{\text{F}}}_{{\text{ACA}}}\left({\varphi }\right) = -0.5 \times {\text{ACA}}\times {\text{cos}}2 \left({\varphi }-{\alpha }\right) \left(0\leq {\varphi } < \pi \right)$$
- The refractive power at an arbitrary angle φ of the ± 0.5 × PCA virtual cross cylinder with an axis angle of β (0 ≤ β < π), corresponding to the angle of the PK_f_’ can be expressed as (Fig. 2b):
  11 $${{\text{F}}}_{{\text{PCA}}}\left({\varphi }\right) = -0.5 \times {\text{PCA}}\times {\text{cos}}2 \left({\varphi }-{\alpha }\right) \left(0\leq {\varphi } < \pi \right)$$
- Then, the refractive power of the TCA at an arbitrary angle φ can be expressed as follows:
  12 $${{\text{F}}}_{{\text{TCA}}}\left({\varphi }\right)= {{\text{F}}}_{{\text{ACA}}}\left({\varphi }\right)+{{\text{F}}}_{{\text{PCA}}}\left({\varphi }\right)\ \left(0\le {\varphi }<\uppi \right)$$

The astigmatic magnitude of the TCA and the axis angle σ (0 ≤ σ < π) corresponding to the angle of the flattest meridian of the TCA can be calculated by substituting σ for φ and solving the following equation:

13 $${{\text{F}}}_{{\text{TCA}}} (\sigma) = {{\text{F}}}_{{\text{ACA}}} (\sigma) + {{\text{F}}}_{{\text{PCA}}} (\sigma) (0 \leq \sigma < \pi)$$

### Calculation of the magnitude and axis of the total corneal astigmatism

Equation (12) is rewritten as the following Eq. (14) using Eqs. (10) and (11).

14 $$\begin{aligned}{{\text{F}}}_{{\text{TCA}}}\left({\varphi }\right)&= {{\text{F}}}_{{\text{ACA}}}\left({\varphi }\right)+{{\text{F}}}_{{\text{PCA}}}\left({\varphi }\right) \\ &=-0.5\times \text{ACA}\times {\text{cos}}2\left({\varphi }-{\alpha }\right)-0.5\times \text{PCA}\times {\text{cos}}2\left({\varphi }-\upbeta \right) \\ &=-0.5\times \left\{\left({\text{ACAsin}}2{\alpha }+{\text{PCAsin}}2\upbeta \right)\,{\text{sin}}2{\varphi }+\left({\text{ACAcos}}2{\alpha }+{\text{PCAcos}}2\upbeta \right)\,{\text{cos}}2{\varphi }\right\}\\ &=-0.5\times \left({\text{a}}\times {\text{sin}}2{\varphi }+{\text{b}}\times {\text{cos}}2{\varphi }\right) \\ &=-0.5\sqrt{{{\text{a}}}^{2}+{{\text{b}}}^{2}}\times {\text{cos}}\left(2{\varphi }-\uptheta \right) \\ a&=\text{ACA}\times {\text{sin}}2{\alpha }+{\text{PCA}}\times {\text{sin}}2\upbeta \\ b&=\text{ACA}\times {\text{cos}}2{\upalpha }+{\text{PCA}}\times {\text{cos}}2\upbeta \\ &\text{Here}, \uptheta \text{ should satisfy the following equations}. \\ \mathrm{sin}\uptheta &=\frac{{\text{a}}}{\sqrt{{{\text{a}}}^{2}+{{\text{b}}}^{2}}} \quad \text{and}\quad \mathrm{cos}\uptheta =\frac{{\text{b}}}{\sqrt{{{\text{a}}}^{2}+{{\text{b}}}^{2}}} \\ \end{aligned}$$

The astigmatic magnitude of the TCA is the difference between the maximum and minimum value of F_TCA_(φ), that is, $\sqrt{{{\text{a}}}^{2}+{{\text{b}}}^{2}}$ is equivalent to twice the amplitude of Eq. (14).

15 $${\text{TCA}}=\sqrt{{{\text{a}}}^{2}+{{\text{b}}}^{2}}=\sqrt{{{\text{ACA}}}^{2}+{{\text{PCA}}}^{2}+2\times {\text{ACA}}\times {\text{PCA}}\times {\text{cos}}\left(2{\alpha }-2\upbeta \right)}$$

The axis angle σ of the TCA is determined as follows:

16 $$\mathrm{tan\uptheta }=\frac{\mathrm{sin\uptheta }}{\mathrm{cos\uptheta }}=\frac{{\text{a}}}{{\text{b}}}\ \left(-\uppi <\uptheta \le\uppi \right)\ \left({\text{Fig}}. 3{\text{a}}\right)$$

In Eq. (14), σ is equal to φ when F_TCA_(φ) is the minimum value as described in Eq. (13). Therefore,

17 $${{\text{F}}}_{{\text{TCA}}}\left(\upsigma \right)=-0.5\sqrt{{{\text{a}}}^{2}+{{\text{b}}}^{2}}\times {\text{cos}}\left(2\upsigma -\uptheta \right)\ \left(0\leq \sigma < \pi \right)$$

18 $$\uptheta ={\text{arctan}}\left(\frac{{\text{a}}}{{\text{b}}}\right)\ \left(-\frac{\uppi }{2} < \theta < \frac{\pi }{2}\right)\ \left({\text{Fig}}. \,3{\text{b}}\right)$$

**Figure 3:**
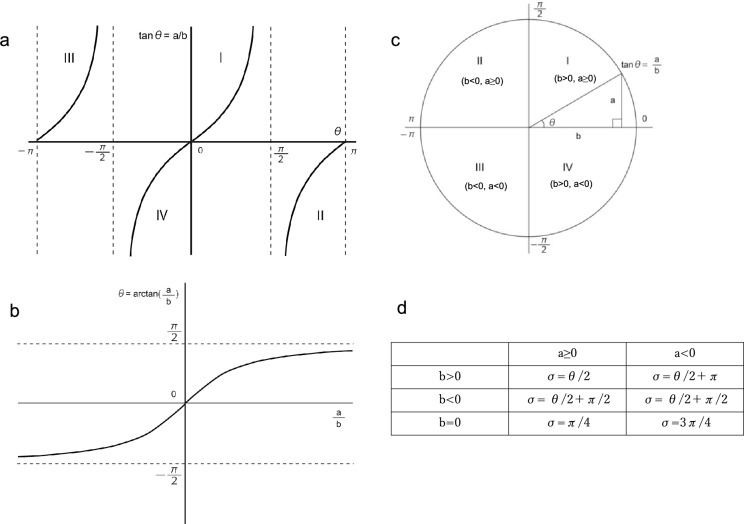
(**a**) Graphical display of the relationship between θ and $\mathrm{tan \theta }$= a/b. a = ACA × sin2α + PCA × sin2β, b = ACA × cos2α + PCA × cos2β; (**b**) Graphical display of the relationship between a/b and θ = arctan(a/b). The natural domain of the arctangent function is – π/2 < θ < π/2. (**c**) Application of the atan2 function. If b > 0, θ is in the first or fourth quadrants and thus within the natural domain of the arctangent function, so θ_ATAN2_ = arctan(a/b) + 0. If b < 0 and a ≥ 0, θ is in the second quadrant, which is beyond the natural domain of the arctangent function, so θ_ATAN2_ = arctan(a/b) + π. If b < 0 and a < 0, θ is in the third quadrant, which is beyond the natural domain of the arctangent function, so θ_ATAN2_ = arctan(a/b) − π. If b = 0 and a > 0, θ_ATAN2_ = π/2. If b = 0 and a < 0, θ_ATAN2_ =  − π/2. (**d**) Axis angle σ of the total corneal astigmatism, which is determined based on the combination of the signs of a and b.

Although the range of θ is − π < θ ≤ π (Fig. 3a), the range is $-\frac{\uppi }{2}<\uptheta <\frac{\uppi }{2}$ here, since this range is the natural domain of the arctangent function (Fig. 3b). Then, the range of θ can be extended to − π < θ ≤ π by applying the atan2 function^16,17^:

19 $$\begin{array}{lll}{\uptheta }_{{\text{ATAN}}2}&={\text{atan}}2\left({\text{a}},\mathrm{ b}\right) &\left(-\uppi <{\uptheta }_{{\text{ATAN}}2}\le\uppi \right) \\&=\text{arctan}\left(\frac{{\text{a}}}{{\text{b}}}\right) &\text{if b}>0 \\ &=\text{arctan}\left(\frac{{\text{a}}}{{\text{b}}}\right)+\pi &\text{if b}<0, \text{a}\ge 0\\ &=\text{arctan}\left(\frac{{\text{a}}}{{\text{b}}}\right)-\pi &\text{if b}<0, \text{a}<0\\ &=\frac{\uppi }{2} &\text{if b}=0, \text{a}>0\\ &=-\frac{\uppi }{2} &\text{if b}=0, \text{a}<0\\ &=\text{undefined} &\text{if b}=0, \text{a}=0\end{array}$$

Equation (17) can be rewritten as the following Eq. (20) using the atan2 function.

20 $${{\text{F}}}_{{\text{TCA}}}\left(\upsigma \right)=-0.5\sqrt{{{\text{a}}}^{2}+{{\text{b}}}^{2}}\times {\text{cos}}\left(2\upsigma -{\uptheta }_{{\text{ATAN}}2}\right)$$

1. If b>0,

21 $$\begin{aligned} {\text{F}}_{{{\text{TCA}}}} \left( {{\upsigma }} \right) & = - 0.5\sqrt {{\text{a}}^{2} + {\text{b}}^{2} } \times \cos \left( {2{{\upsigma }} - {\text{arctan}}\left( {\frac{{\text{a}}}{{\text{b}}}} \right)} \right) \\ & = - 0.5\sqrt {{\text{a}}^{2} + {\text{b}}^{2} } \times \cos \left( {2{{\upsigma }} - {{\uptheta }}} \right)\\ {\text{When}} & \cos \left( {2{{\upsigma }} - {{\uptheta }}} \right) = 1, {\text{F}}_{{{\text{TCA}}}} \left( {{\upsigma }} \right){\text{ is the minimum}}. \\ \end{aligned}$$

If a≥0 and b>0,

$-\frac{\uppi }{2}<2\upsigma -\uptheta <2\uppi$ is derived from $0\le 2\upsigma <2\pi \,\mathrm{and }\,0\le\uptheta <\frac{\uppi }{2}$, since θ is in the first quadrant, as shown in Fig. 3c. By solving Eq. (21):

$$2\upsigma -\uptheta =0$$

22 $$\therefore\upsigma =\frac{\uptheta }{2}$$

Then, if a<0 and b>0,

$0<2\upsigma -\uptheta <\frac{5}{2}\uppi$ is derived from $0\le 2\upsigma <2\pi \,\mathrm{and }-\frac{\uppi }{2}<\uptheta <0$, since θ is in the fourth quadrant, as shown in Fig. 3c. By solving Eq. (21):

$$2\upsigma -\uptheta =2\uppi$$

23 $$\therefore\upsigma =\frac{\uptheta }{2}+\uppi$$

2. If b < 0 and a ≥ 0,

24 $$\begin{aligned}{{\text{F}}}_{{\text{TCA}}}\left(\upsigma \right)&=-0.5\sqrt{{{\text{a}}}^{2}+{{\text{b}}}^{2}}\times {\text{cos}}\left(2\upsigma -\left({\text{arctan}}\left(\frac{{\text{a}}}{{\text{b}}}\right)+\uppi \right)\right)\\ &=-0.5\sqrt{{{\text{a}}}^{2}+{{\text{b}}}^{2}}\times {\text{cos}}\left(2\upsigma -\left(\uptheta +\uppi \right)\right) \\ &=+0.5\sqrt{{{\text{a}}}^{2}+{{\text{b}}}^{2}}\times {\text{cos}}\left(2\upsigma -\uptheta \right) \\ &\text{When cos}\left(2\upsigma -\uptheta \right)=-1, {{\text{F}}}_{{\text{TCA}}}\left(\upsigma \right) \text{ is the minumun}.\end{aligned}$$

$-\uppi <2\upsigma -\uptheta <\frac{3}{2}\uppi$ is derived from $0\le 2\upsigma <2\pi \,\mathrm{and }\frac{\uppi }{2}<\uptheta \le\uppi$, since θ is in the second quadrant, as shown in Fig. 3c. By solving Eq. (24):

$$2\upsigma -\uptheta =\uppi$$

25 $$\therefore\upsigma =\frac{\uptheta }{2}+\frac{\uppi }{2}$$

3. If b < 0 and a < 0,

26 $$\begin{aligned} {\text{F}}_{{{\text{TCA}}}} \left( {{\upsigma }} \right) & = - 0.5\sqrt {{\text{a}}^{2} + {\text{b}}^{2} } \times \cos \left( {2{{\upsigma }} - \left( {{\text{arctan}}\left( {\frac{{\text{a}}}{{\text{b}}}} \right) - {{\uppi }}} \right)} \right) \\ & = - 0.5\sqrt {{\text{a}}^{2} + {\text{b}}^{2} } \times \cos \left( {2{{\upsigma }} - \left( {{{\uptheta }} - {{\uppi }}} \right)} \right) \\ & = + 0.5\sqrt {{\text{a}}^{2} + {\text{b}}^{2} } \times \cos \left( {2{{\upsigma }} - {{\uptheta }}} \right)\\ {\text{When}} & \cos \left( {2{{\upsigma }} - {{\uptheta }}} \right) = - 1,{\text{F}}_{{{\text{TCA}}}} \left( {{\upsigma }} \right){\text{ is the minumun}}. \\ \end{aligned}$$

$\frac{\uppi }{2}<2\upsigma -\uptheta <3\uppi$ is derived from $0\le 2\upsigma <2\pi \,\mathrm{and }-\uppi <\uptheta <-\frac{\uppi }{2}$, since θ is in the third quadrant, as shown in Fig. 3c. By solving Eq. (26):

$$2\upsigma -\uptheta =\uppi$$

27 $$\therefore\upsigma =\frac{\uptheta }{2}+\frac{\uppi }{2}$$

4. If b = 0 and a > 0,

28 $$\begin{aligned} & {\text{F}}_{{{\text{TCA}}}} \left( {{\upsigma }} \right) = - 0.5\sqrt {{\text{a}}^{2} + {\text{b}}^{2} } \times \cos \left( {2{{\upsigma }} - \frac{{{\uppi }}}{2}} \right) \\ & {\text{When}}\cos \left( {2{{\upsigma }} - \frac{{{\uppi }}}{2}} \right) = 1,{\text{F}}_{{{\text{TCA}}}} \left( {{\upsigma }} \right){\text{is the minimum}}. \\ \end{aligned}$$

By solving Equation (28):

$${\text{sin}}2\sigma =1,\ 0\leq 2\upsigma <2\uppi$$

$$2\sigma =\frac{\pi }{2}$$

29 $$\therefore\upsigma =\frac{\uppi }{4}$$

5. If b = 0 and a < 0,

30 $$\begin{aligned} & {\text{F}}_{{{\text{TCA}}}} \left( {{\upsigma }} \right) = - 0.5\sqrt {{\text{a}}^{2} + {\text{b}}^{2} } \times \cos \left( {2{{\upsigma }} + \frac{{{\uppi }}}{2}} \right) \\ & {\text{When}}\cos \left( {2{{\upsigma }} + \frac{{{\uppi }}}{2}} \right) = 1,{\text{F}}_{{{\text{TCA}}}} \left( {{\upsigma }} \right){\text{ is the minimum}}. \\ \end{aligned}$$

By solving Eq. (30):

$${\text{sin}}2\upsigma =-1,\ 0\le 2\upsigma <2\uppi$$

$$2\upsigma =\frac{3}{2}\uppi$$

31 $$\therefore\upsigma =\frac{3}{4}\uppi$$

In summary, the axis angle σ of the TCA is determined based on the combination of the signs of a and b, as shown in Fig. 3d. Finally, since σ is the angle of the axis of the flattest meridian of the TCA, the magnitude and direction of the TCA can be expressed as follows:

32 $$\mathrm{TCA\ }{@\ }\sigma \pm \frac{\pi }{2}\ \left(0\leq\sigma \pm \frac{\pi }{2}<\pi \right)$$

$$\mathrm{where TCA}=\sqrt{{{\text{ACA}}}^{2}+{{\text{PCA}}}^{2}+2\times {\text{ACA}}\times {\text{PCA}}\times {\text{cos}}\left(2{\alpha }-2\upbeta \right)}$$.

In the + cylinder form, the notation for the TCA is:

33 $$\mathrm{Sph }-0.5\times \mathrm{TCA }=\mathrm{Cy }+\mathrm{TCA\ Ax\ \sigma }$$

In the − cylinder form, the notation is:

34 $$\mathrm{Sph }+0.5\times {\text{TCA}}=\mathrm{Cy }-\mathrm{TCA\ Ax\ \sigma }\pm \frac{\pi }{2}\ \left(0\leq\sigma \pm \frac{\pi }{2}<\pi \right)$$

To determine the magnitude and astigmatic axis of the TCA, the notation (33) can be applied since the sign of Cy is positive and the angle of the axis is that of the flattest meridian. To correct the TCA, we need to implement the following spherocylindrical lenses in the TK plane.

In the − cylinder form, the lens is:

35 $$\mathrm{Sph }+0.5\times \mathrm{TCA }=\mathrm{Cy }-\mathrm{TCA\ Ax\ \sigma }$$

In the + cylinder form, the lens is:

36 $$\mathrm{Sph }-0.5\times \mathrm{TCA }=\mathrm{Cy }+\mathrm{TCA\ Ax\ \sigma }\pm \frac{\pi }{2}\ \left(0\leq\sigma \pm \frac{\pi }{2}<\pi \right)$$

### Verification experiment with the Jackson cross cylinder

**Figure 4:**
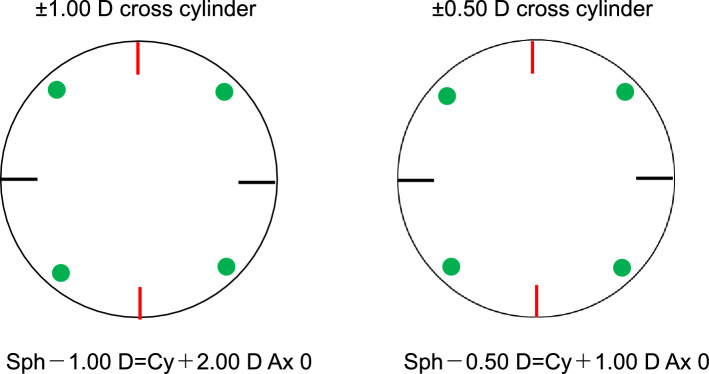
The two Jackson cross cylinders used for the verification experiment: ± 1.00 D (S – 1.00 D = C + 2.00 D Ax 0) and ± 0.50 D (S – 0.50 D = C + 1.00 D Ax 0). *D* diopter, *Ax* axis.

To evaluate the performance of the virtual cross cylinder method, a verification experiment was performed. Since it is impossible to make the virtual cross cylinder, the Jackson cross cylinder was used as an alternative. Two Jackson cross cylinders of ± 1.00 D and ± 0.50 D were prepared, and the axes of the + cylinder, corresponding to the black marks in Fig. 4, were designated as the axes of the cross cylinders. The ± 1.00 D cross cylinder with axis angle α and the ± 0.50 D cross cylinder with axis angle β can be expressed as Sph − 1.00 D = Cy + 2.00 D Ax α and Sph − 0.50 D = Cy + 1.00 D Ax β, respectively. The two stacked Jackson cross cylinders were mounted in a trial frame with angle scales, each axis was incrementally rotated, and the resultant power and axis of the combined cross cylinders were measured with an automatic lensmeter (LM-1800PD, NIDEK). The measurements were performed for all 324 combinations of axis angles α and β from 0 to 170 at 10-degree intervals. To compare the measured astigmatic axes and magnitudes with those calculated by the virtual cross cylinder method, scatter plots using Cartesian coordinates were generated to visually confirm the agreement between the measured and calculated values, and the results were further evaluated using Spearman’s rank correlation coefficients. The intraclass correlation coefficient (ICC) and the Bland − Altman plots cannot be determined because of the nonnormal distribution. All analyses were performed using BellCurve for Excel version 4.04 (Social Survey Research Information Co., Ltd.).

## Results

**Figure 5:**
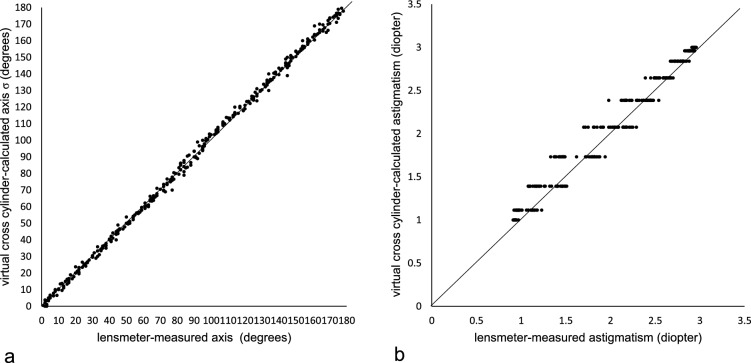
Plots of the lensmeter-measured and virtual cross cylinder method-calculated values with two Jackson cross cylinders of ± 1.00 D and ± 0.50 D. (**a**) Astigmatic axis. (**b**) Astigmatic magnitude. The oblique line in the graph is y = x. *D* diopter.

Figure 5 shows scatter plots of the astigmatic axis (Fig. 5a) and astigmatic magnitude (Fig. 5b) resulting from the combination of the two Jackson cross cylinders with ± 1.00 D and ± 0.50 D to compare the values measured with the lensmeter with the values calculated by the virtual cross cylinder method. The astigmatic axes and magnitudes converge on the line y = x (x: measured values, y: calculated values). Based on the 324 pairs of data, Spearman’s rank correlation coefficient was *r* = 0.9993 (*p* < 0.001) for the astigmatic axis. Similarly, Spearman’s rank correlation coefficient was *r* = 0.9833 (*p* < 0.001) for the astigmatic magnitude; thus, the results were highly correlated. Compared with the astigmatic axis results, the wider range of convergence on the line y = x for the astigmatic magnitude likely occurs because the combination of the Jackson cross cylinders, which have thick lenses, cannot be conducted on the same plane as the virtual TK plane for the virtual cross cylinders. Moreover, the Jackson cross cylinders have different power profiles from the virtual cross cylinder.

## Discussion

I developed the virtual cross cylinder method for calculating the total corneal astigmatism by combining the anterior corneal astigmatism and posterior corneal astigmatism on a virtual TK plane, corresponding to the secondary principal plane of the cornea. The results of the verification experiment using Jackson cross cylinders showed that the values calculated by the virtual cross cylinder method were consistent with those measured by the lensmeter.

**Figure 6:**
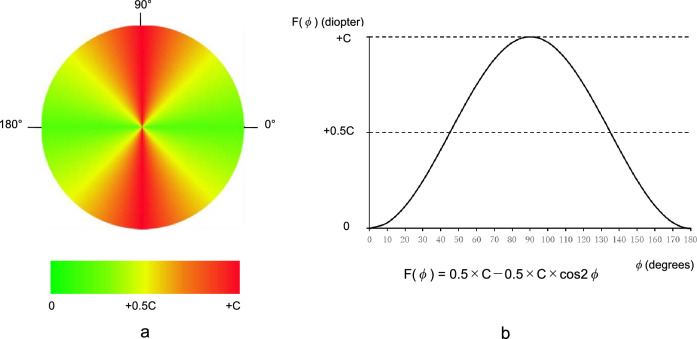
A spherocylindrical lens of Cy + C Ax 0, depicting the power profile with a color coded map (**a**) and a graphical display (**b**) of the power F(φ). *C* power of the spherocylindrical lens.

Historically, astigmatism has been analyzed based on power vector analysis^18–22^. This method is very complex since it addresses the spherical and astigmatic components of spherocylindrical lenses at the same time. In contrast, the virtual cross cylinder method analyzes only the astigmatic component and not the spherical component on a spherical equivalent plane by using trigonometric functions. Furthermore, Eq. (37) (Appendix in Supplementary Equations) in the paper by Furlan et al.^23^ is the same as Eq. (9), (10), and (11) in the present study. Equation (37) defines the astigmatic component derived from Eq. (38) (Supplementary Equations) which describes the dioptric power of a spherocylindrical lens at the off-axis meridian. The similarity between the formulas occurs because the trigonometric double-angle formula gives Eq. (39) (Supplementary Equations). There has been much discussion on the derivation and results of the sine-squared expression for spherocylindrical lenses^24–27^. Although the above results have not been noted in previous works, Eq. (37) is equivalent to the power F(φ) at an arbitrary angle φ of the virtual cross cylinder. Hence, the sine-squared expression of the spherocylindrical lens is identical to the expression of the combination of the virtual cross cylinder and the spherical component with 0.5 × C. In other words, the virtual cross cylinder has only the astigmatic component of the spherocylindrical lens, with the spherical component removed. Furthermore, consider the spherocylindrical lens. Consider a spherocylindrical lens of Cy + C Ax 0 with a refractive power of zero (green) at its astigmatic axis (Fig. 6a). The refractive power F(φ) of this lens at an arbitrary angle φ is expressed as Eq. (40) (Supplementary Equations). Using the trigonometric double-angle formula, this equation can be transformed into Eq. (41) (Supplementary Equations), which is equivalent to the sine-squared expression of a spherocylindrical lens, as described above.

Several vector analysis techniques for astigmatism have been derived based on the abovementioned traditional power vector analysis method^28–33^. These methods have been applied to calculate the TCA^12–15^. However, the results of these studies using vector analysis methods to combine anterior corneal astigmatism and posterior corneal astigmatism have not been verified experimentally, as was performed in this study. The results of the vector analysis methods should be verified through “ex vivo” verification experiments, even if these methods are widely used and empirically correct. The present study is the first to confirm that the virtual cross cylinder method is valid for combining anterior corneal astigmatism and posterior corneal astigmatism. Moreover, Abulafia et al.^13^ suggested that a thick lens formula should be employed to calculate the net corneal power for each meridian. This approach is consistent with the virtual cross cylinder method, which calculates the total corneal astigmatism on the TK plane based on Gaussian optics. Furthermore, Ferreira et al.^15^ and Savini et al.^34^ reported that toric IOL power calculations based on the predicted posterior corneal astigmatism rather than the measured astigmatism yielded better refractive outcomes. Their results might be attributed to the calculation of the TCA via vector analysis without using a thick lens formula, and hence, reevaluation of these results with the virtual cross cylinder method on the TK plane is of great interest.

In this study, I set the reference axis of the virtual cross cylinder at the meridian with the least refractive power (shown in blue in Fig. 1a) and expressed the refractive power at an arbitrary angle φ through the Eq. (9). However, the reference axis can be arbitrarily chosen. For example, when the axis at which the power is zero, that is, a neutral refractive power, as designated in green in Fig. 1a, is chosen as the reference axis, the refractive power at an arbitrary angle φ is defined as Eq. (42) (Supplementary Equations). In contrast, when the axis at which the power is + 0.5 × C, that is, the maximum refractive power, as designated in red in Fig. 1a, is chosen as the reference axis, the refractive power at an arbitrary angle φ is defined as Eq. (43) (Supplementary Equations). These equations can also be used to calculate the TCA using trigonometric functions and the atan2 function. This study adopted the meridian with the least refractive power − 0.5 × C as the reference axis to ensure consistency with the sine-squared expression.

The atan2 function was first introduced in computer science^17^ and has since been applied in other fields. With this function, the natural domain of the arctangent function, $-\frac{\uppi }{2}<\uptheta <\frac{\uppi }{2}$, is extended to − π < θ_ATAN2_ ≤ π, which was used to calculate the TCA in this study. Since the notation of the atan2 function is yet to be mathematically established, either atan2(y, x) or atan2(x, y) can be used. Care should be taken when using Microsoft Excel, which adopts the format atan2(x, y). When Excel is used, the procedure is defined using Eq. (20) and Eqs. (44) and (45) (Supplementary Equations). Additional data for the verification experiments using the virtual cross cylinder method and the vector analysis method are shown in the corresponding Excel files (Supplementary Information).

The virtual cross cylinder method can also be applied to a combination of spherocylindrical lenses. As described above, the refractive power at an arbitrary angle φ of a spherocylindrical lens Cy + C Ax 0 is expressed as Eq. (40) (Supplementary Equations). Given that the power of a spherocylindrical lens 1 of Cy + C_1_ Ax α is Eq. (46) (Supplementary Equations), and the power of a spherocylindrical lens 2 of Cy + C_2_ Ax β is Eq. (47) (Supplementary Equations), the refractive power of the combined spherocylindrical lens 1 + 2 at an arbitrary angle φ is calculated as Eq. (48) (Supplementary Equations). This is exactly the same format as Eq. (14) shown in the Methods section, except for the spherical component of 0.5 × (C_1_ + C_2_). The results of the verification experiments using two spherocylindrical lenses confirmed that the astigmatic magnitude and axis value calculated with the virtual cross cylinder method were consistent with those measured by the lensmeter.

In addition, the virtual cross cylinder method can be applied to subtraction of astigmatism, or the calculation of surgically induced astigmatism (SIA). Cornea-based SIA is caused by incisions during cataract surgery and ablations during corneal refractive surgery, and lens-based SIA is caused by toric intraocular lens implantation. By applying the virtual cross cylinder method on the secondary principal plane of a given optical system, Eq. (49) (Supplementary Equations) can be defined, where F_PRE_(φ) is the refractive power at an angle φ for the preoperative astigmatism, F_SIA_(φ) is the refractive power at an angle φ for the SIA, and F_POST_(φ) is the refractive power at an angle φ for the postoperative astigmatism. Then, F_SIA_(φ) can be calculated by solving the given equations. When calculating the F_SIA_(φ), the magnitude and direction of the SIA are similar to those determined by the equations described by Jaffe and Clayman^35^, which Naeser^33^ introduced as Eqs. (50) and (51) (Supplementary Equations). However, it is highly likely that Eq. (51) generates incorrect results because it does not use the atan2 function. On the other hand, Naeser^33^ adjusted the arctangent function in Eq. (52) (Supplementary Equations) in his paper by adding the integer *p*, which somehow adjusted the limit of the natural domain of the arctangent function. However, Eq. (52)^33^ cannot handle scenarios in which the denominator KP(φ + 45) is 0, requiring the addition of a very small value to the denominator, such as 10^–10^, as shown by Holladay et al.^28^. In contrast, the virtual cross cylinder method using the atan2 function addresses this limit with a simple and straightforward approach.

The advantages of the virtual cross cylinder method are as follows: the method is easy to intuitively understand since it calculates the astigmatic axis and magnitude mathematically using familiar trigonometric functions; Eq. (9), which defines F(φ) =  − 0.5 × C × cos2φ at an arbitrary angle φ, can be universally applied to express regular astigmatic components, demonstrating the versatility of the virtual cross cylinder method; and the method is consistent with the sine-squared expression, as described above, and is applicable to both spherocylindrical lenses and the cross cylinder. On the other hand, there are several limitations to this study. First, the virtual cross cylinder method assumes regular astigmatism, in which the principal meridians are perpendicular to each other. However, as corneal topography maps show, the cornea has an irregular astigmatic component. In extreme cases such as irregular astigmatism, the extraction of the regular astigmatic component by the Fourier transform might be required before the method can be applied. Second, since the virtual cross cylinder cannot be physically produced, the Jackson cross cylinder was used as an alternative in the verification experiment. The results of the verification experiments showed good agreement between the measured and calculated values. A study on the combination of anterior corneal astigmatism and posterior corneal astigmatism in the real cornea is currently being performed. Finally, this study addresses the TCA calculation for an individual case; on the other hand, for aggregate data analysis, it remains to be determined whether previously published and well-known methods such as the double-angle plot^36^ and bivariate polar value analysis^37^ are suitable for the virtual cross cylinder method. Alternatively, from a different viewpoint, directional statistics^38^ may be appropriate since the virtual cross cylinder method is based on trigonometric functions.

In conclusion, I developed the virtual cross cylinder method, a novel technique for calculating the total corneal astigmatism on the TK plane. This method is expected to tremendously contribute to the fields of ophthalmology and optometry, including refractive surgery, cataract surgery, contact lens and spectacle prescription, and other optical design methods. Finally, I hope that the virtual cross cylinder method will be added to the principles of optics.

### Supplementary Information

Supplementary Information 1.

Supplementary Information 2.

Supplementary Information 3.

## Data Availability

The datasets used and/or analyzed during the current study available from the corresponding author on reasonable request.
