# Supplementary material for: Calculation of the total corneal astigmatism using the virtual cross cylinder method on the secondary principal plane of the cornea
Source: Sci Rep. 2024 Feb 26;14:4611. doi: 10.1038/s41598-024-55154-x (PMC11310508; doi:10.1038/s41598-024-55154-x)
Supplement: Supplementary file 1 — Supplementary Information 1. [file 41598_2024_55154_MOESM1_ESM.docx]

**Appendix**

$$C_{\theta}=-0.5\times C\times\cos\left( 2\theta-2\alpha\right) (37)$$

$$P_{\varphi}=S+C\times\sin^{2}\left( \varphi-\theta\right) (38)$$

$${{C\times sin}^{2}\left( \varphi-\theta\right)=0.5\times C\times\left( 1-\cos2\left( \varphi-\theta\right) \right) \atop=0.5\times C-0.5\times C\times\cos2\left( \varphi-\theta\right)} (39)$$

$$F\left( \varphi\right)=0.5\times C-0.5\times C\times\cos2\varphi\left( Fig. 6b \right) (40)$$

$$F\left( \varphi\right)=C\times\sin^{2}\varphi(41)$$

$$F\left( \varphi\right)=+0.5\times C\times\sin2\varphi(42)$$

$$F\left( \varphi\right)=+0.5\times C\times\cos2\varphi(43)$$

$\theta_{ATAN2}=ATAN2\left( b, a \right) (44)$

When cos(2σ – θ_ATAN2_) = 1, F_TCA_(σ) is at its minimum.

$-\pi\leq2\sigma-\theta_{ATAN2}<3\pi$ is derived from $0\leq2\sigma<2\pi and -\pi<\theta_{ATAN2}\leq\pi$.

By solving Equation (20):

$2\sigma-\theta_{ATAN2}=0 or 2\pi$

$$\therefore\sigma=\frac{\theta_{ATAN2}}{2}\mathrm{or}\frac{\theta_{ATAN2}}{2}+\pi\left( 0\leq\sigma<\pi\right) (45)$$

$$F_{1}\left( \varphi\right)=0.5\times C_{1}-0.5\times C_{1}\times\cos2\left( \varphi-\alpha\right) (46)$$

$$F_{2}\left( \varphi\right)=0.5\times C_{2}-0.5\times C_{2}\times\cos2\left( \varphi-\beta\right) \left( 47 \right)$$

$${F_{1+2}\left( \varphi\right)=F_{1}\left( \varphi\right)+F_{2}\left( \varphi\right) \atop\begin{aligned} =0.5\times\left( C_{1}+C_{2} \right)-0.5\sqrt{a^{2}+b^{2}}\times\cos\left( 2\varphi-\theta\right) \\ a=C_{1}\times\sin2\alpha+C_{2}\times\sin2\beta\\ b=C_{1}\times\cos2\alpha+C_{2}\times\cos2\beta\\ \sin\theta=\frac{a}{\sqrt{a^{2}+b^{2}}}\mathrm{and}\cos\theta=\frac{b}{\sqrt{a^{2}+b^{2}}} \end{aligned}} (48)$$

$F_{\mathrm{PRE}}\left( \varphi\right)+F_{\mathrm{SIA}}\left( \varphi\right)=F_{\mathrm{POST}}\left( \varphi\right)$ (49)

$$M_{\mathrm{SIA}}^{2}=M_{\mathrm{preop}}^{2}+M_{\mathrm{postop}}^{2}-2M_{\mathrm{preop}}\times M_{\mathrm{postop}}\times\cos\left( 2\emptyset_{\mathrm{postop}}-2\emptyset_{\mathrm{preop}} \right) (50)$$

$$\emptyset_{\mathrm{SIA}}=\frac{1}{2}\times arctan\left[ \frac{M_{\mathrm{postop}}\times\sin\left( 2\emptyset_{\mathrm{postop}} \right)-M_{\mathrm{preop}}\times\sin\left( {2\emptyset}_{\mathrm{preop}} \right)}{M_{\mathrm{postop}}\times\cos\left( 2\emptyset_{\mathrm{postop}} \right)-M_{\mathrm{preop}}\times\cos\left( 2\emptyset_{\mathrm{preop}} \right)} \right] (51)$$

$$\alpha=arctan\left( \frac{M-KP\left( \Phi\right)}{\mathrm{KP}\left( \Phi+45 \right)} \right)+p\times180+\Phi(52)$$
